# How is the positive deviance approach applied within healthcare organizations? A systematic review of methods used

**DOI:** 10.1186/1472-6963-14-S2-P7

**Published:** 2014-07-07

**Authors:** Ruth Baxter, Ian Kellar, Natalie Taylor, Rebecca Lawton

**Affiliations:** 1 Institute of Psychological Sciences, University of Leeds, Leeds, UK; 2 Yorkshire Quality and Safety Research Group, Bradford Institute for Health Research, Bradford, UK; 3 Centre for Clinical Governance Research, University of New South Wales, Sydney, Australia

## Background

The positive deviance approach assumes that solutions to problems faced by a community already exist. Despite experiencing similar constraints as others, ‘positive deviants’ succeed through their deviant behaviour. Originating within international public health, positive deviance has increasingly been applied to healthcare and a four stage process has been proposed to do this. Positive deviants are firstly identified using routinely collected data then their success strategies are explored. These strategies are then tested in a more representative sample and disseminated to the community. The quality of current healthcare applications however varies and different study designs and methods are used at each stage of the process. This review aims to investigate the extent to which these stages are adopted in healthcare research.

## Materials and methods

The review explored definitions of positive deviance, study designs and methods used, quality of research, level of analyses conducted and organizations or employees involvement within the research. The search strategy was applied across seven electronic databases and reference list/ citation searches were conducted for included articles. Studies were included if they used positive deviance within a healthcare organization, reported primary research and were peer reviewed. Data extraction focused on the methods used at each of the four stages of positive deviance. Quality assessment was conducted using the QATSDD and all extraction was second reviewed for accuracy and completeness. Data synthesis was conducted using the UK Economic and Social Research Council guidance on narrative synthesis.

## Results

The search strategy identified 564 articles excluding duplicates. 37 articles, representing 26 individual projects met the full inclusion criteria. Positive deviance is most commonly applied to reduce healthcare associated infections and research has most frequently been conducted within North America. Research focuses on stages 1 and 2; identifying positive deviants and the strategies used to succeed. Very little research tests or disseminates these strategies. Disparate approaches, study designs and methods are used within research with little involvement from the organisation and staff.

## Conclusions

This is the first systematic review of positive deviance applications within healthcare organisations. It highlights the approaches flexibility, relevance to a range of quality improvement issues and use in identifying practical and sustainable solutions. Recommendations relate to the study designs and methods used, practicalities of conducting the approach within clinical contexts and the increased role organisations and employees could have in positive deviance research.

